# Prevalence and associated factors of thrombocytopenia among neonates in Northwest Amhara region comprehensive specialized hospitals, Ethiopia: a cross sectional study

**DOI:** 10.1038/s41598-025-93042-0

**Published:** 2025-04-12

**Authors:** Asnake Tadesse Abate, Gezahagn Demsu Gedefaw, Chanyalew Worku Kassahun, Mengistu Mekonnen Kelkay

**Affiliations:** 1 Department of Neonatal Health Nursing, School of Nursing, College of Medicine and Health Sciences, and Specialized Hospital, University of Gondar, Gondar, Ethiopia; 2 Department of Medical Nursing, School of Nursing, University of Gondar, Gondar, Ethiopia; 3 Department of Pediatrics and Child Health Nursing, School of Nursing, University of Gondar, Gondar, Ethiopia

**Keywords:** Neonate, Thrombocytopenia, Hospitals, Northwest Amhara, Ethiopia, Medical research, Paediatric research

## Abstract

Thrombocytopenia is a common hematologic concern in neonates and can lead to severe complications, including significant bleeding particularly intracranial hemorrhage. This risk is especially critical in preterm infants or those with other coexisting conditions. While neonatal thrombocytopenia has been studied in some countries, there is limited data and understanding about its prevalence, and associated factors in resource-limited countries especially in Ethiopia. So this study aimed to assess the prevalence and factors associated with thrombocytopenia among neonates admitted to the neonatal intensive care unit of Northwest Amhara Region Comprehensive Specialized Hospitals in Northwest Ethiopia, 2022. Institution-based cross-sectional study design was conducted from October 05 to November 03, 2022, and a systematic random sampling technique was used to select 423 study participants. Data were collected by interviewing mothers and reviewing neonates’ medical records. Data were entered into Epi-Data version 4.6.0 and exported to STATA version 14 for analysis. Both bivariable and multivariable logistic regression models were used for analysis. P-value of less than 0.05 and odds ratio with 95% CI was used to declare the presence of association. A total of 415 neonate-mother pairs were involved with a response rate of 98.1%. The prevalence of thrombocytopenia among neonates admitted to the neonatal intensive care unit Is found to be 26.02%; [95% CI (22.01–30.48%)]. In the multivariable analysis severe pre-eclampsia (AOR = 2.84 95% CI 1.29–6.27), prolonged rupture of membrane (AOR = 2.85 95% CI 1.21–6.72), neonatal sepsis (AOR = 6.50 95% CI 3.58–11.79), perinatal asphyxia (AOR = 4.15 95% CI 1.97–8.76), and necrotizing enterocolitis (AOR = 3.71 95% CI 1.68–8.19) were significant factors to thrombocytopenia. To decrease the occurrence of neonatal thrombocytopenia better to give special attention and priority to neonates diagnosed with sepsis, perinatal asphyxia, necrotizing enterocolitis, and mothers who had prolonged rupture of membrane or severe pre-eclampsia.

## Background

Neonatal thrombocytopenia (NT) is defined as a platelet count below the lower limit of the normal range, although clinically dangerous bleeding is not seen until counts drop well below 50, 000 per microliter^1,2^. It is classified into two based on the onset of time to early-onset thrombocytopenia (occurs within 72 h after birth) and late-onset thrombocytopenia (occurs after 72 h of birth)^3–5^.

Studies showed that the prevalence of NT was 14% in the Balkans^6^, and 24–68.4% in Pakistan^7,8^. In Africa, the magnitude of NT ranged from 12.4 to 53%^7,9–11^ whereas, in Ethiopia, its prevalence in preterm neonates with early onset sepsis was 48.5%^12^. It is among the most common hematological problems observed in neonates admitted to the neonatal intensive care unit (NICU), particularly affecting sick newborns and preterm infants during the neonatal period^1,13^.

Studies conducted in South Asia showed that 12.6-14.5% of neonates died from thrombocytopenia and 9-28.2% developed intraventricular hemorrhage (IVH)^6,14–16^. Several studies show that IVH is the most serious complication and is more common in infants with NT (10–20%), especially for extremely low birth weight neonates or preterm neonates^2,3,17^. Neonates with thrombocytopenia are also more likely to experience various types of abnormal bleeding, such as gastrointestinal, pulmonary, and cutaneous bleeding^2,17^. Despite the government’s priority issue of reducing neonatal mortality, it has increased from 29 to 33 per 1000 live births from 2016 to 2019^18,19^. Meanwhile, NT is recognized as one of the significant contributors to neonatal morbidity and mortality. A systematic review and meta-analysis studies revealed that thrombocytopenia is one of the causes of failure of ductus arteriosus to spontaneously close in preterm newborns, which resulted in neonatal morbidity and mortality^20,21^.

The development of thrombocytopenia in the intensive care unit typically signifies the complication of the co-morbidities process and needs prolonged hospitalization for recuperation. In addition, it requests high cost that leads to economic burdens on the family, society, and health care systems. To alleviate the problem and increase the survival of neonates, the world health organization (WHO) recommends the ministry of health to expand the quality of services for neonates by strengthening newborn care and maternal obstetric health programs^9,22,23^.

Various studies suggest that small for gestational age (SGA), pregnancy-induced hypertension, prematurity, perinatal asphyxia (PNA), sepsis, polycythemia, gestational diabetes mellitus (GDM), and placental insufficiency were factors significantly associated with early-onset thrombocytopenia whereas late-onset thrombocytopenia is typically greater and frequently secondary to neonatal sepsis or necrotizing enterocolitis (NEC)^2,4,24,25^.

Despite NT being so common, little attention is given to the assumption that it has resolved spontaneously even though having significant health problems. However, if it is not identified early and treated effectively, it might cause fatal consequences or internal bleeding^5,26,27^. Despite this, there is limited information that shows the prevalence and factors significant with NT in Ethiopia. Therefore, determining the prevalence and its associated factors can be used as a piece of evidence for the concerned stakeholders to address the problem and the potential factors for neonatal thrombocytopenia.

## Methods and materials

### Study design, period, and area

A multicenter institution-based cross-sectional study design was conducted from October 05 to November 03, 2022, in Northwest Amhara regional State comprehensive specialized hospitals. The study was conducted in five Comprehensive Specialized Hospitals (University of Gondar, Felege-Hiwot, Tibebe Ghion, Debre Tabor, and Debre-Markos). Those hospitals have special units; like NICU, and the major services in NICU include general neonatal care services, routine prescription of complete blood count, blood exchange transfusion, phototherapy, and ventilation support. The average annual admission of neonates was 4,560 (2,562 inborn) at University of Gondar Comprehensive Specialized Hospital, 1,836 (1,003 inborn) at Felege Hiwot Comprehensive Specialized Hospital, 1,920 (1,085 inborn) at Tibebe Ghion Comprehensive Specialized Hospital, 1,560 (847 inborn) at Debre Tabor Comprehensive Specialized Hospital and 1,692 (953 inborn) at Debre Markos Comprehensive Specialized Hospital.

### Population

The source populations were all mothers with their neonates that were admitted to the NICU of Northwest Amhara region comprehensive specialized hospitals, and the study populations were all neonate-mother pairs in the study area during the study period. Neonates who had no platelet count as part of their lab investigation and mothers who were critically ill or treated in other wards/ intensive care units were excluded.

### Sample size determination, sampling procedure, and study variables

The sample size was calculated using the single population proportion formula by considering an assumption *Zα*/2 = 1.96, proportion was considered to be 50%, and d = the margin error between the sample and the population, which is 5%.

By adding a 10% non-response rate the final sample size became 423. The study was conducted in the Northwest Amhara region’s five comprehensive specialized hospitals. The average baseline data at one-year admission data were taken from each hospital to consider the available data. Then allocation of the sample size to each hospital was made based on the average number of neonate admission per month. The overall sample was taken proportionally from each hospital and a systematic random sampling technique (sampling interval ≈ 3) was used to select the sample participants. The first participant was selected randomly by manually lottery method at the beginning of the data collection time.

### Variables of the study

#### Dependent variable

Neonatal thrombocytopenia (Yes/No).

#### Independent variables

##### Socio-demographic characteristics of the mother and the neonate

Maternal age, residence, educational status, marital status, occupational status, and sex of the baby.

#### Maternal obstetric-related variables

Parity, antenatal care (ANC) follow-up, place of delivery, mode of delivery, hepatitis B virus infection, SPE, eclampsia, GDM, antepartum hemorrhage (APH), premature rupture of membranes (ROM) and prolonged ROM.

#### Neonatal-related variables

Gestational age, birth weight, number of index pregnancy, weight for gestational age and prolonged not feeding orally (NPO).

#### Neonatal clinical co-morbidity variables

Sepsis, NEC, PNA, jaundice, neonatal surgical management, polycythemia, meconium aspiration syndrome (MAS), respiratory distress syndrome (RDS), and anemia.

### Operational definition

Thrombocytopenia: A platelet count of less than 150,000 per microliter of blood regardless of the gestational age^28^.

Prolonged NPO: A neonate who is NPO for greater than 24 h^29^.

Prolonged ROM: Rupture of membrane more than 18 h^30^.

Low birth weight: An infant born weighing a birth of less than 2500 g^31^.

Prematurity: A neonate is born before 37 weeks of pregnancy have been completed^32^.

Premature ROM: a rupture of membranes before labor starts^30^.

Neonatal surgical management: Neonates who had surgical management for tracheoesophageal fistula or gastroschisis or omphalocele or anorectal malformations^33^.

### Data collection tool, procedure and quality control

The data collection tool was adapted by reviewing related kinds of literature and adopted from validated standards like the Ethiopian demographic health survey checklists. It was prepared in English and translated into the Amharic language, then retranslated to English by language experts and health professionals to check for consistency and flow, while for clinical variables (chart review checklists) English version was developed. Before starting data collection, the tool was evaluated and commented on by a research expert for its validity. After verbal and written informed consent was obtained from the mothers, data were collected by interviewing mothers and reviewing neonates’ medical records. Three supervisors: Sr. Tigst Asres (BSc neonatal nurse), Mr. Awugichew Esubalew (BSc neonatal nurse) and Mrs. Rahel Asres (MSc pediatrics nurse) and data collectors: Mr. Molla Asfaw (BSc neonatal nurse), Asnakew Tigabu (BSc neonatal nurse), and Mr. Misganew Tesfaye (BSc neonatal nurse) were involved in the data collection process. A 5% pretest was conducted at the Woldiya Comprehensive Specialized Hospital before the actual data collection for the validity of the instrument or questionnaire.

### Data processing and analysis

Data were checked, coded, and entered into Epi-Data version 4.2.0 and exported to STATA version 14 for analysis. The outcome variable was dichotomized and coded as 0 and 1, representing those who are not thrombocytopenic and thrombocytopenic respectively. Pearson rank chi-square assumption fulfillment was checked for categorical variables. A bivariable logistic regression analysis was performed to find the association of each independent variable with the outcome variables. All variables with a significant association in the bivariable logistic regression analysis (*p* < 0.2) were entered into the final multivariable logistic regression analysis to control confounders and identify the independent effects of different factors on the occurrence of thrombocytopenia. Adjusted odds ratio (AOR) with a 95% confidence interval (CI) was calculated, and a p-value of < 0.05 was considered statistically significant. The goodness of model fitness was tested by the Hosmer and Lemeshow test and the model were adequate. The variance inflation factor was used to test multicollinearity (VIF = 1.41).

### Ethical approval and consent to participate

Ethical approval (Ref. No. 035/2022) was obtained from the ethical review committee of the Institutional Review Board (IRB) of the University of Gondar for the Amhara Public Health Institute. A formal letter of permission was obtained from the Amhara Public Health Institute for each hospital. Finally, written consent was obtained from the mothers after a thorough explanation of purposes, benefits, information confidentiality, and the voluntary nature of participation. All methods were performed in accordance with the declarations of Helsinki and relevant guidelines and regulations. The names and/or identification numbers of the study participants were not recorded on the data collection tool. All information was kept completely private and utilized exclusively for the objectives of this study.

## Result

### Socio-demographic and behavioral characteristics

A total of 415 neonate-mother pairs were included in the study with a response rate of 98.1%. The mean age of the mothers was 26.8 (± 5.1 SD) years ranging from 18 to 40 years, and most (86.5%) mothers belonged to the age groups of 20–35 years. Regarding the occupational status of the mothers, nearly 44% of mothers were housewives, and more than half of neonates (57.1%) were females (Table 1).

**Table 1:** Socio-demographic characteristics of the mothers with their neonates admitted to NICU of Northwest Amhara region comprehensive specialized hospitals, Northwest Ethiopia, 2022 (*n* = 415).

Variable	Category	Frequency	Percentage (%)
Age of the mothers	< 20	19	4.6
	20–35	359	86.5
	> 35	37	8.9
Residence	Urban	225	54.2
	Rural	190	45.8
Maternal educational status	No formal education	48	11.6
	Primary school	89	21.5
	Secondary school	101	24.4
	Diploma and above	177	42.7
Maternal occupation	Government employee	94	22.7
	Private employee	62	14.9
	Merchant	60	14.5
	Daily labor	18	4.3
	Housewife	181	43.6
Marital status	Single	16	3.9
	Married	337	81.2
	Divorced	21	5.1
	Widowed	41	9.9
Sex of the newborn	Male	178	42.9
	Female	237	57.1

### Maternal obstetric history

Most (98.6%) mothers attended ANC follow-up for the index pregnancy, and out of those, 55.3% had four and above visits. Most (91.8%) mothers delivered at health institutions, and more than half (58.3%) of mothers gave birth via spontaneous vaginal delivery (SVD) (Table 2).

**Table 2:** Maternal obstetric history of neonates in NICU of Northwest Amhara region comprehensive specialized hospitals, Northwest Ethiopia, 2022 (*n* = 415).

Variables	Category	Frequency	Percentage (%)
Parity	Primi para	155	37.4
	Multi-para	260	62.7
ANC visit	Yes	409	98.6
	No	6	1.6
Number of ANC visits (*n* = 409)	1–3	183	44.7
	≥ 4	226	55.3
Place of delivery	Home	34	8.2
	Health institution	381	91.8
Mode of delivery	SVD	242	58.3
	Assisted vaginal delivery	34	8.2
	Cesarean section	139	33.5

### Maternal obstetric complications

This study finding revealed that 12.1% of mothers had experienced SPE. In addition, 13.0% of mothers had premature ROM and 9.4% of mothers had prolonged ROM (Table 3).

**Table 3:** History of obstetric complications of mothers whose neonates are admitted to NICU of Northwest Amhara region comprehensive specialized hospitals, Northwest Ethiopia, 2022 (*n* = 415).

Variable	Category	Frequency	Percentage (%)
SPE	Yes	50	12.1
	No	365	87.9
Prolonged_ROM	Yes	39	9.4
	No	376	90.6
Premature_ROM	Yes	54	13.0
	No	361	86.9
GDM	Yes	29	6.9
	No	386	93.0
APH	Yes	42	10.1
	No	373	89.9
Eclampsia	Yes	25	6.0
	No	390	93.9
HBsAg	Yes	19	4.6
	No	396	95.4
Others	Yes	27	6.5
	No	388	93.5

*APH* antepartum hemorrhage, *GDM* gestational diabetes mellitus, *HBsAg* hepatitis B virus surface antigen, *ROM* rupture of membrane and *SPE* severe preeclampsia, Other include: HIV Infection, prolonged labor, obstructed labor, and chorioamnionitis.

### Neonatal-related factors

Out of the total newborn babies, more than two-thirds (68.7%) of neonates were born at term, and 67.2% of neonates had a birth weight of 2500 g and above. The mean age and weight of neonates at the time of birth were 37.6 (± 2.6 SD) weeks and 2717.6 (± 841.8 SD) grams, respectively (Table 4).

**Table 4:** Neonatal-related factors of neonates admitted to NICU of Northwest Amhara region comprehensive specialized hospitals, Northwest Ethiopia, 2022 (*n* = 415).

Variables	Category	Frequency	Percentage (%)	
Birth weight	< 2500 g	136	32.8	
	≥ 2500 g	279	67.2	
Gestational age	< 37 weeks	130	31.3	
	≥ 37 weeks	285	68.7	
Number of the index pregnancy	Singleton	354	85.3	
	Twin	53	12.8	
	Triple or more	8	1.9	
Weight for gestational age	AGA	350	84.3	
	SGA	51	12.3	
	LGA	14	3.4	
Did the neonate NPO	Yes	78	18.8	
	No	337	81.2	
If yes how long is NPO (*n* = 78)	≤ 24 h	2	2.6	
	> 24 h	76	97.4	

*NPO*   not per oral (no feeding), *AGA*  appropriate for gestational age, *SGA* small for gestational age, *LGA* large for gestational age.

### Neonatal clinical co-morbidities

Neonatal clinical co-morbidities during admission were reviewed from the medical record of the neonates. Out of 415 reviewed charts, neonates admitted to NICU regarding co-morbidities more than one-third of the neonates (36.9%) had sepsis, 22.4% had jaundice, and 20% had MAS (Table 5).

**Table 5:** Neonatal clinical co-morbidities of neonates admitted to NICU of Northwest Amhara region comprehensive specialized hospitals, Northwest Ethiopia, 2022 (*n* = 415).

Variable	Category	Frequency	Percentage (%)
Sepsis	Yes	153	36.9
	No	262	63.1
PNA	Yes	59	14.2
	No	356	85.9
NEC	Yes	48	11.6
	No	367	88.4
Surgical management	Yes	18	4.3
	No	397	95.7
Jaundice	Yes	93	22.4
	No	322	77.6
MAS	Yes	83	20.0
	No	332	80.0
RDS	Yes No	48 367	11.6 88.4
Anemia	Yes No	80 335	19.3 80.7
Polycythemia	Yes	44	10.6
	No	371	89.4
Others	Yes	47	11.3
	No	368	88.7

*PNA* perinatal asphyxia, *NEC* necrotizing enterocolitis, *MAS* meconium aspiration syndrome and *RDS* respiratory distress syndrome.

Other include: pancytopenia, infant of a diabetic mother, DIC, and aspiration pneumonia.

### Prevalence of neonatal thrombocytopenia

The prevalence of thrombocytopenia among neonates admitted to the NICU of Northwest Amhara region comprehensive specialized hospitals was found to be 26.02% with 95% CI (22.01, 30.48%).

### Factors associated with neonatal thrombocytopenia

From the multivariable logistic regression analysis SPE, prolonged ROM, neonatal sepsis, PNA, and NEC were statistically significant with neonatal thrombocytopenia.

Neonates born from SPE mothers were 2.8 times more likely to have thrombocytopenia as compared to neonates born from normotensive mothers [(AOR: 2.84, 95% CI (1.29 6.27)]. Similarly, neonates born from a mother who had prolonged ROM were approximately 3 times more likely to develop thrombocytopenia than those delivered from a mother who had not prolonged ROM diagnosed [(AOR: 2.85, 95% CI (1.21 6.72]. The odds of thrombocytopenia were 6.5 times higher among neonates who had sepsis as compared to their counterparts [AOR: 6.50; 95%CI (3.58, 11.79)]. The neonates with PNA were approximately 4.2 times more likely to have thrombocytopenia, as compared to those neonates who had not PNA diagnosed [AOR: 4.15; 95%CI (1.97 8.76)]. In the same way, the odds of thrombocytopenia were 3.7 times more in neonates who had NEC than those neonates who had no NEC diagnosed [AOR: 3.71, 95% CI (1.68 8.19)]. (Table 6)

**Table 6:** Regression table consists of both binary logistic regression and multivariable logistic regression of thrombocytopenia among neonates admitted to NICU in Northwest Amhara region comprehensive specialized hospitals, Northwest Ethiopia, 2022.

Variables	Categories	Thrombocytopenia		COR (95% CI)	AOR (95% CI)
		Yes	No		
SPE	No Yes	**81** (22.2%) **27** (54%)	**284** (77.8%) **23** (46%)	1.00 4.12 (2.24 7.56)*	1.00 **2.84**(**1.29 6.27**)**
Prolonged_ ROM	No Yes	**87** (23.1%) **21** (53.9%)	**289** (76.9%) **18** (46.1%)	1.00 3.88 (1.98 7.60)*	1.00 2.85 (1.21 6.72)**
Maternal age	20–35 < 20 > 35	**90** (25.1%) **8** (42.1%) **10** (27%)	**269** (74.9%) **11** (57.9%) **27** (73%)	1.00 2.17 (0.85 5.57)* 1.11 (0.52 2.38)	1.00 2.64 (0.96 12.19) 1.63 (0.59 4.48)
Marital status	Married	**86** (25.5%)	**251**(74.5%)	1.00	1.00
	Single	**3** (18.8%)	**13** (81.2%)	0.67 (0.19 2.42)	0.31(0.06 1.65)
	Widowed	**15** (36.6%)	**26** (63.4%)	1.68 (0.85 3.34)*	1.51(0.59 3.89)
	Divorced	**4** (19.1%)	**17** (80.9%)	0.69 (0.22 2.09)	0.67 (0.18 2.49)
Eclampsia	No Yes	**94** (24.1%) **14** (56%)	**296** (75.9%) **11** (44%)	1.00 4.01 (1.76 9.13)*	1.00 2.48 (0.82 7.48)
Sepsis	No Yes	**31** (11.8%) **77** (50.3%)	**231** (88.2%) **76** (49.7%)	1.00 7.55 (4.62 12.33)*	1.00 6.50 (3.58 11.79)**
PNA	No Yes	**75** (21.1%) **33** (55.9%)	**281** (78.9%) **26** (44.1%)	1.00 4.76 (2.68 8.44)*	1.00 4.15 (1.97 8.76)**
NEC	No Yes	**78** (21.3%) **30** (62.5%)	**289** (78.8%) **18** (37.5%)	1.00 6.18 (3.27 11.66)*	1.00 3.71 (1.68 8.19)**
Birth weight	≥ 2500 gms < 2500 gms	**48** (17.2%) **60** (44.1%)	**231** (82.8%) **76** (55.9%)	1.00 3.79 (2.39 6.02)*	1.00 1.97 (0.78 4.99)
Gestational Age	≥ 37wks < 37wks	**57** (20%) **51** (39.2%)	**228** (80%) **79** (60.8%)	1.00 2.58 (1.64 4.08)*	1.00 2.24 (0.91 5.49)
Weight for gestational age	AGA SGA LGA	**76** (21.7%) **27** (52.9%) **5** (35.7%)	**274** (78.3%) **24** (47.1%) **9** (64.3%)	1.00 4.06(2.21 7.43)* 2.00 (0.65 6.15)	1.00 1.36 (0.56 3.29) 2.67 (0.43 11.39)

*Statistically significant by COR at *p*-value < 0.2, **Statistically significant by AOR at *p*-value < 0.05.

Significant values are in (bold).

## Discussion

In this study, the prevalence of neonatal thrombocytopenia was 26.02% [95% CI (22.01%, 30.48%)], which was in line with previous studies conducted in Pakistan (24%)^8^, India (25.45% and 29%)^2,4^, and Iran (28.5%)^34^.

However, this result was higher than the findings from Tunisia (12.4%)^35^, South Africa (15.6%)^11^, and Nepal (18%)^5^. The discrepancy might be due to the difference in the study settings like the quality of NICU, which meant infection prevention practice differences in hospitals. In Ethiopia hospitals, the infection prevention practice was 52.2%^36^ whereas in Nepal was 76.2%^37^. Additionally, the possible justification might be being a single-centric study while the current study is multi-institutional-based.

On the other hand, this study also had a lower prevalence than other studies carried out in Ethiopia (48.5%)^38^, Egypt (38.18%)^39^, Nigeria (53%)^40^, India (36.1%)^41^, and Pakistan (68.24%)^42^. This variation might be due to the study population differences between the previous Ethiopia study, which focused only on preterm neonates with sepsis^38^, the Indian study, which focused solely on neonates born from pregnancy-induced hypertension^41^, and the Pakistan study, which focused exclusively on neonatal sepsis^42^ whereas all neonates, regardless of gestational age, were used in the current study. Another possible justification may be the sample size they used is much lower than the current study^39,40^.

Multivariable logistic regression analysis showed that SPE, prolonged ROM, neonatal sepsis, PNA, and NEC were factors significantly associated with NT.

The present study identified that the odds of having thrombocytopenia among neonates who were delivered to SPE mothers were 2.8 times higher compared with those neonates who were delivered from normotensive mothers. This finding was supported by studies from Egypt^39^, Nepal^5^, Iran^34^, India^43^, and South Asia^8^. This might be because preeclampsia is a multi-system disorder associated with adaptive changes in the fetal circulation and causes a marked imbalance in the haemostatic system of the mother and the neonate end up with thrombocytopenia. Since SPE causes placental insufficiency results in a direct depressant effect on fetal megakaryocytopoiesis, which is the cellular developmental process before the release of platelets into the circulation. On the other hand, it also causes maternal low platelet which gives rise to antibodies against her platelets resulting in passive transmission of those antibodies from mother to fetus through the placenta and attacking the fetus platelet end up with thrombocytopenia^25,44^. Additionally, chronic exposure to increased levels of erythropoietin in the fetus due to fetal hypoxia secondary to preeclampsia may also lead to thrombocytopenia by suppressing the megakaryocytic cell line which may lead to decreased platelet production^45^.

The odds of thrombocytopenia were approximately 3 times higher among neonates who were born to mothers diagnosed with prolonged ROM compared with those neonates born to mothers who had no prolonged ROM. This finding was supported by finding in the United Kingdom^46^. It might be because a woman with prolonged rupture of membranes is at risk of intra-amniotic infection (perinatal infection), which is considered one of the most conditions for early-onset neonatal thrombocytopenia^25^. The activation of reticuloendothelial cells and subsequent platelet removal are caused by injury to endothelial cells, which is the most likely cause of platelet count reduction^47^ .

According to the current study, neonates with sepsis were 6.5 times more likely to be thrombocytopenic as compared to neonates without sepsis, which was in line with studies in Ethiopia^38,48^, Egypt^39,49^, Nigeria^40^, Tunisia^50^, Nepal^5^, South Asia^15^, and UK^27^. Due to the fact that sepsis increases the risk of thrombocytopenia by destruction and the consumption of platelet results in thrombocytopenia. Since thrombocytopenia is a common and multifactorial phenomenon occurring during sepsis^3,25^. The other possible reason is that bacteria may cause endothelial damage leading to platelet adhesion and aggregation or may bind directly to platelets leading to aggregation and accelerated clearance from circulation and it is also a highly specific marker of fungal sepsis^42,51^. In addition, the possible justification may be that sepsis-induced thrombocytopenia is caused by a decrease in platelet synthesis in the bone marrow^52^.

Those neonates who had PNA had approximately 4.2 times higher odds for thrombocytopenia when compared with neonates without PNA. This is consistent with other studies carried out in Nigeria^40^, Nepal^5^, Iran^34^, India^43^, South Asia^15^, and Europe^13^. It might be because PNA decreases platelet production and it is considered one of the most common causes of early-onset thrombocytopenia. Moreover, PNA is also associated with disseminated intravascular coagulation, which may cause platelet activation and consumption^25,53^. Additionally, neonates who suffer from issues like perinatal asphyxia can become seriously sick and develop thrombocytopenia as a result of platelet consumption from a complication^54^.

Neonates who had NEC had 3.7 times higher odds for thrombocytopenia than neonates without NEC diagnosed. This is supported by studies carried out in the USA^55^, and Europe^13^. Fifty to ninety-five% of all neonates with NEC develop thrombocytopenia within 24–72 h and the primary mechanism for thrombocytopenia is widely believed to be increased platelet destruction in the endothelium of vessels that supply blood to areas of the bowel in the early stages of necrosis^56,57^. In addition, the possible justification may be because of the following two reasons: the first one is due to stimulation of endothelial cells and macrophages to release inflammatory cytokines, which, along with thromboplastin released from gangrenous bowel, can increase platelet activation and aggregation in the microvasculature^56^ and the second one is elevated thrombopoietic response is due to increased levels of platelet factor 4, which is released from activated platelets and is a potent inhibitor of megakaryocytopoiesis^58^.

This study has some limitations that should be considered. The cross-sectional nature of the study did not tell the temporal relationship among variables. Data lacks maternal medication and TORCH infection history that may affect NT, because of not recorded in the neonatal medical chart.

## Conclusions

The main obstetric complications of mothers of neonatal admission in the study area were premature ROM followed by SPE, and the majority of neonates were born at term. The prevalence of neonatal thrombocytopenia among neonates admitted to the neonatal intensive care unit was found to be high. SPE, prolonged ROM, neonatal sepsis, PNA, and NEC were factors significantly associated with the prevalence of neonatal thrombocytopenia. To minimize the burden of neonatal thrombocytopenia, particular interest should be given to neonates diagnosed with sepsis, PNA, NEC, and mothers who had prolonged ROM or SPE. Future researchers will better do longitudinal research that can identify the temporal relationship to make the inference.

List of Tables.

## Data Availability

The datasets generated during the current study are not publicly available due to confidentiality issues. But data will be available upon reasonable request from the corresponding author.

## Abbreviations

AGA: Appropriate for gestational age
ANC: Antenatal care
AOR: Adjusted odd ratio
APH: Antepartum hemorrhage
CI: Confidence interval
COR: Crude odd ratio
GDM: Gestational diabetes mellitus
HBsAg: Hepatitis B virus surface antigen
ICH: Intracranial hemorrhage
IVH: Intraventricular hemorrhage
LBW: Low birth weight
LGA: Large for gestational age
MAS: Meconium aspiration syndrome
NEC: Necrotizing enterocolitis
NICU: Neonatal intensive care unit
NPO: Not per oral
NT: Neonatal thrombocytopenia
PNA: Perinatal asphyxia
RDS: Respiratory distress syndrome
ROM: Rupture of membrane
SD: Standard deviation
SGA: Small for gestational age
SPE: Severe preeclampsia
SVD: Spontaneous vaginal delivery
